# Enteric Bacterial Pathogens in Children with Diarrhea in Niger: Diversity and Antimicrobial Resistance

**DOI:** 10.1371/journal.pone.0120275

**Published:** 2015-03-23

**Authors:** Céline Langendorf, Simon Le Hello, Aissatou Moumouni, Malika Gouali, Abdoul-Aziz Mamaty, Rebecca F. Grais, François-Xavier Weill, Anne-Laure Page

**Affiliations:** 1 Epicentre, Paris, France; 2 Institut Pasteur, Unité des Bactéries Pathogènes Entériques, Centre National de Référence des *Escherichia coli*, *Shigella* et *Salmonella*, Paris, France; 3 Epicentre, Niamey, Niger; The Ohio State University, UNITED STATES

## Abstract

**Background:**

Although rotavirus is the leading cause of severe diarrhea among children in sub-Saharan Africa, better knowledge of circulating enteric pathogenic bacteria and their antimicrobial resistance is crucial for prevention and treatment strategies.

**Methodology/Principal Findings:**

As a part of rotavirus gastroenteritis surveillance in Maradi, Niger, we performed stool culture on a sub-population of children under 5 with moderate-to-severe diarrhea between April 2010 and March 2012. *Campylobacter*, *Shigella* and *Salmonella* were sought with conventional culture and biochemical methods. *Shigella* and *Salmonella* were serotyped by slide agglutination. Enteropathogenic *Escherichia coli* (EPEC) were screened by slide agglutination with EPEC O-typing antisera and confirmed by detection of virulence genes. Antimicrobial susceptibility was determined by disk diffusion. We enrolled 4020 children, including 230 with bloody diarrhea. At least one pathogenic bacterium was found in 28.0% of children with watery diarrhea and 42.2% with bloody diarrhea. Mixed infections were found in 10.3% of children. EPEC, *Salmonella* and *Campylobacter* spp. were similarly frequent in children with watery diarrhea (11.1%, 9.2% and 11.4% respectively) and *Shigella* spp. were the most frequent among children with bloody diarrhea (22.1%). The most frequent *Shigella* serogroup was *S*. *flexneri* (69/122, 56.5%). The most frequent *Salmonella* serotypes were Typhimurimum (71/355, 20.0%), Enteritidis (56/355, 15.8%) and Corvallis (46/355, 13.0%). The majority of putative EPEC isolates was confirmed to be EPEC (90/111, 81.1%). More than half of all Enterobacteriaceae were resistant to amoxicillin and co-trimoxazole. Around 13% (46/360) *Salmonella* exhibited an extended-spectrum beta-lactamase phenotype.

**Conclusions:**

This study provides updated information on enteric bacteria diversity and antibiotic resistance in the Sahel region, where such data are scarce. Whether they are or not the causative agent of diarrhea, bacterial infections and their antibiotic resistance profiles should be closely monitored in countries like Niger where childhood malnutrition pre-disposes to severe and invasive infections.

## Introduction

Although diarrhea mortality rate among children under 5 years of age in sub-Saharan Africa has been decreasing annually by about 4% since 2000, mortality remains high [1]. Of the estimated 3.6 million child deaths in 2010, 12% were attributed to diarrheal diseases. The incidence of childhood diarrhea in Africa has also decreased from 4.2 to 3.3 episodes per child-year from 1990 to 2010, but sub-Saharan Africa still accounts for one third of diarrheal episodes yearly (500 million of 1.7 billion worldwide), with the highest incidence among children 6–11 months of age [2].

While rotavirus is the leading cause of severe diarrhea among children under 5 [3–5], with about 30% of diarrhea cases being rotavirus positive in sub-Saharan Africa [6], many other viruses, parasites and bacteria cause serious diarrheal morbidity and mortality but are not vaccine preventable [7–12]. Among bacterial etiologies, typical enteropathogenic *Escherichia coli* (EPEC) are associated with a higher risk of mortality in infants 0–11 months of age with moderate-to-severe diarrhea and *Shigella* is the first or second cause of moderate-to-severe diarrhea among children 1 to 5 years old [7,13].

The Sahel (an ecoregion lying between the Sudanian savanna to the south and the Sahara desert to the north) bears the burden of some of the highest rates of diarrhea, malnutrition and malaria among children under 5 [1]. The vicious cycle involving diarrheal disease and malnutrition places this population at higher risk of morbidity and mortality [7,14,15]. Moreover, some diarrhea-causing agents like non-typhoidal *Salmonella* (NTS) were found to be the dominant contributor to invasive bacterial disease with high mortality in Africa [16–18]. Several studies in the Sahelian region showed that multi-drug resistant organisms have been emerging over the last decades, with the general spread of resistance to amoxicillin, co-trimoxazole and chloramphenicol [19–25]. Resistance to fluoroquinolones and extended-spectrum cephalosporins (ESCs) in Enterobacteriaceae have also been reported to a lower extent [26–29]. Data on the prevalence and antimicrobial resistance of the main circulating enteropathogenic bacteria remain limited in the Sahel and in Niger in particular, due to the lack of laboratory facilities. Yet, better knowledge of circulating endemic and epidemic enteric pathogens and their antimicrobial resistance is crucial for prevention, including vaccines against *Shigella* and diarrheagenic *E*. *coli* [30,31] and treatment strategies with antibiotics.

Here, we describe the main enteric and possibly pathogenic bacteria isolated from the stools of children under 5 with moderate-to-severe diarrhea included in rotavirus gastroenteritis surveillance in Maradi, Niger between April 2010 and March 2012.

## Materials and Methods

### Study sites

Surveillance was set up as described previously [32] in the region of Maradi, located approximately 500 km away from the capital city Niamey. One regional hospital and 10 health centers were included in four different health districts: Maradi (1 regional hospital), Madarounfa (3 health centers), Aguié (3 health centers) and Guidan Roumdji (4 health centers). After an initial period of one year of surveillance, surveillance was interrupted in the health district of Aguié. The analysis presented here was restricted to a 24-month period from April 2010 to March 2012.

### Study population

Children were included in the general rotavirus surveillance study if they were aged 0 to 59 months, consulting at a study site with watery diarrhea and signs of moderate or severe dehydration, and if the parent or legal guardian accepted participation in the study. Children with reported bloody diarrhea were included during the second year [32]. A sub-sample of the children included in the general rotavirus surveillance was selected to participate in the sub-study on bacterial etiology of diarrhea. These children were: the first child included daily with watery diarrhea at each health center; the first 2 children included daily with watery diarrhea at the regional hospital; all included children with reported bloody diarrhea and producing stool specimens.

### Data and specimen collection

A standardized questionnaire was used to collect socio-demographic characteristics (sex, age), clinical presentation (dehydration, fever, vomiting), medical care (rehydration treatment, hospitalization, inclusion in a nutritional program) and outcomes.

At the health centers, stool specimens were collected using sterile plastic containers and systematically tested for rotavirus using Vikia Rota-Adeno rapid test (bioMérieux, Marcy l’Etoile, France) [32]. Stool samples were then transferred onto Cary-Blair medium (Oxoid, Basingstoke, UK) using sterile swab and stored at +2–8°C until transported in cold containers to the Epicentre laboratory in Maradi city every other day. At the Maradi regional hospital, we maintained the fresh stools at +2–8°C before transfer to the laboratory within 1 hour after sampling.

### Stool culture, bacterial identification and antimicrobial susceptibility testing

The bacterial pathogens sought included *Salmonella* spp, *Shigella* spp, EPEC, and, starting from July 2010, *Campylobacter* spp. Upon arrival at the laboratory, swabs from the Cary-Blair tubes or fresh stools were plated onto desoxycholate citrate lactose media (DCL, Bio-Rad, Marnes-la-Coquette, France), Hektoen media (HK, Bio-Rad), Butzler media (Oxoid) and Muller-Kauffmann broth (MK, AES Chemunex, Combourg, France). DCL, HK and MK were incubated at 37°C in normal atmosphere. Butzler media were incubated for 48h in microaerophilic atmosphere using atmospheric generator (bioMérieux). After 18h incubation, 10μL of MK broth were inoculated onto Chromagar *Salmonella* media (ChromAgar, Paris, France) and incubated for an additional 18–24 h. After incubation, suspected colonies of *Salmonella* and *Shigella* spp. were submitted to metabolic identification using API strips (bioMérieux) and partially serogrouped by slide agglutination using polyvalent *Salmonella* O-typing antisera (Bio-Rad). From DCL media, between 1 and 10 lactose-fermenting colonies were picked and tested by slide agglutination with EPEC O-typing antisera (Bio-Rad). Twelve EPEC serogroups were targeted by the four polyvalent antisera used: I (O111, O55, O26), II (O86, O119, O127), III (O125, O126, O128), and IV (O114, O124, O142). These serogroups corresponded to those recognized as being EPEC by the World Health Organization (WHO) in 1987, with the exception of O124 which was replaced by O158 in the WHO definition [33]. Reactive colonies were subsequently isolated and confirmed as *E*. *coli* using API strips. *Campylobacter* isolates were identified by colony aspect on Butzler media, Gram stain examination, oxidase (+) and catalase (+). The sodium hippurate hydrolysis test was then performed to differentiate *C*. *jejuni* from *C*. *coli*.

Antimicrobial susceptibility of each isolate was determined by the disk diffusion method on Mueller- Hinton agar (+5% sheep blood for *Campylobacter* spp) as recommended by the Antibiogram Committee of the French Society for Microbiology (CA-SFM) [34]. The panel of the 13 antimicrobials tested (Bio-Rad) was amoxicillin (AMX, 25μg), amoxicillin-clavulanic acid (AMC, 20/10μg), cefalotin (CF, 30μg), cefotaxime (CTX, 30μg), ceftazidime (CAZ, 30μg), cefepime (FEP, 30μg), imipenem (IPM, 10μg), nalidixic acid (NA, 30μg), ciprofloxacin (CIP, 5μg), co-trimoxazole (trimethoprim-sulfamethoxazole, SXT, 1,25/23,75μg), gentamicin (GM, 15μg), amikacin (AN, 30μg), and erythromycin (for *Campylobacter* spp. only, E, 15UI). Extended-spectrum β-lactamase (ESBL) phenotype was assessed by using the double-disk synergy (DDS) method [34] on isolates displaying resistance or intermediate resistance to ESCs. The isolates displaying resistance to ciprofloxacin by disk diffusion were confirmed by determination of the minimal inhibitory concentration (MIC) using Etest strips (bioMérieux). CA-SFM breakpoints were used for disk diffusion and the European Committee on Antimicrobial Susceptibility Testing (EUCAST) breakpoints were used for MIC. *E*. *coli* strain CIP 76.24 (ATCC 25922) was used as a quality control strain.

### Typing of Shigella and Salmonella

*Salmonella* (n = 370) and *Shigella* (n = 138) isolates were stored at Epicentre laboratory then sent to the French National Reference Center for *E*. *coli*, *Shigella* and *Salmonella* (FNRC-ESS), Institut Pasteur, Paris. Species identification was confirmed using conventional methods and serotyping was done by slide agglutination assays using a complete set of antisera allowing recognition of all described *Salmonella* [35] and *Shigella* [36] serotypes. Typing antisera were purchased from Bio-Rad, Eurobio (Les Ulis, France), Statens Serum Institute (Copenhagen, Denmark), Sifin (Berlin, Germany) or were homemade at the FNRC-ESS. Two serotype Kentucky isolates were typed by multilocus sequence typing (MLST) as described previously [37].

### Characterization of EPEC isolates

All putative EPEC isolated from rotavirus-positive children (n = 37) and a random sample of putative EPEC isolated from rotavirus-negative children in 2010 (n = 74) were sent to the FNRC-ESS for confirmation and serotyping. All isolates were subjected to the following biochemical tests: motility, glucose (acid and gas), H_2_S, indole production, lysine decarboxylase, ornithine decarboxylase, glycerol, lactose, d-mannitol, and sorbitol. DNA was extracted from all phenotypically confirmed *E*. *coli* isolates with the InstaGene matrix kit (Bio-Rad). PCR was used to detect the *uidA* gene encoding the beta-glucuronidase; thus confirming the assignment of the isolates to *E*. *coli* and to detect several virulence genes associated with EPEC and other diarrheagenic *E*. *coli*. The targeted virulence genes were (i) *eae* (for EPEC attaching and effacing), which encodes intimin, an outer membrane adhesin essential for the intimate attachment of the EPEC or enterohemorrhagic *E*. *coli* (EHEC) to enterocytes, (ii) *bfpA* (for bundle-forming pilus) carried by the EPEC adherence factor (EAF) plasmid found in typical EPEC, (iii) *stx1* and *stx2* encoding Shiga toxins, markers of EHEC, (iv) EHEC-*hlyA* encoding the hemolysin of EHEC, (v) in case of *eae* negativity, *aggR*, a transcriptional activator of aggregative adherence fimbriae expression in enteroaggregative *Escherichia coli* (EAEC), (vi) and in case of *eae* and a*ggR* negativity, *lt* and *st* genes of enterotoxigenic *E*. *coli* (ETEC) and *ial* and *ipah* genes of enteroinvasive *E*. *coli* (EIEC). The different primers and basic amplification conditions are described in S1 Table [38–47]. All *E*. *coli* isolates that contained at least one virulence gene were serogrouped by slide agglutination using at first polyvalent I (O26, O44, O114, O125, O142, O158), II (O55, O86, O91, O111, O119, O126, O127, O128), III (O25, O78, O103, O118, O124, O145, O157, O164) and A (O1, O2, O18, O78) antisera from Sifin; then if needed (i.e., in case of an agglutination with a polyvalent antiserum) the corresponding monovalent antisera, also from Sifin. Not serotypable *E*. *coli* isolates (rough phenotype due to the complete or partial loss of lipopolysaccharide and leading to autoagglutination or no agglutination with the panel of antisera) were O-typed by a molecular method based on the analysis of the O-antigen gene cluster (*rfb* restriction fragment length polymorphism [*rfb*-RFLP]) after DNA extraction with the InstaGene matrix kit (Bio-Rad) [48].

### Data analysis

Double data entry was done using EpiData 3.1 (EpiData, Odense, Denmark), and data analysis using Stata 12.1 (College Station, Texas, USA). Severe diarrhea was defined using the 20-point Vesikari scale with a score ≥11 [49]. We performed a weighted analysis by month and district of inclusion to take into account the selection process when estimating the proportion and seasonality of the bacterial pathogens. For *Salmonella* and *Shigella* spp., only the isolates that were confirmed at FNRC-ESS are considered in this analysis.

### Ethical Considerations

The study protocol was approved by the National Ethical Committee of Niger’s Ministry of Public Health (Ref n° 02/2009/CCNE) and by the Comité de Protection des Personnes, Ile-de-France XI, France. Participation was voluntary. An informed consent statement was read aloud in the local dialect before consenting parent or legal guardian signed or fingerprinted (procedures approved by both ethical committees). All children received treatment free of charge.

## Results

### Characteristics of the study population

In total, 8993 children were included in the rotavirus surveillance study from April 2010 to March 2012. Of these, we excluded 117 because of a delay between admission and testing of more than 3 days, 4 without clinical signs of dehydration, and 37 who sought care ≥ 14 days after onset of diarrhea. The 8835 remaining comprised 8566 children with watery diarrhea, of which 3790 (44.3%) were selected for the bacteriological study; and 269 with bloody diarrhea, of which 230 (85.5%) were also included in the bacteriological study.

Due to the selection process of children for the bacteriological study (i.e. inclusion of the first child per day in all health centers and first 2 children per day at Maradi hospital), children included in Maradi district (i.e. at the Maradi regional hospital), hospitalized children and intravenous treatment are over-represented compared to the initially enrolled population (Table 1). Other characteristics, such as sex, age, presence of fever, inclusion in a nutritional program and mortality were similar to the initial population (Table 1). Children with bloody diarrhea showed significantly less vomiting and a lower proportion of severe diarrhea according to the Vesikari score, compared to those with watery diarrhea (Table 1).

**Table 1:** Socio-demographic and clinical characteristics of children with watery or bloody diarrhea, included in the bacteriology sub-study.

	****Watery diarrhea (N = 3790)****		****p**** *	****Bloody diarrhea (N = 230)****		****p**** ‡
	n	%		n	%	
District of inclusion			<0.001			<0.001
Maradi	658	17.4		37	16.1	
Madarounfa	1201	31.7		54	23.5	
Guidan Roumdji	1482	39.1		139	60.4	
Aguié	449	11.8		0	0.0	
Socio-demographic						
Male	2103	55.5	0.34	125	54.4	0.75
Age, median [IQR]	9	[7–12]		12	[9–19]	
Clinical characteristics						
Severe dehydration	581	15.3	0.11	44	19.1	0.12
Fever	852	22.5	0.53	62	27.0	0.12
Vomiting	2461	65.0	0.006	105	46.1	<0.001
Vesikari score ≥ 11	2759	72.9	0.54	128	55.7	<0.001
Hospitalisation and outcome						
IV treatment	447	11.8	<0.001	21	9.1	0.22
Hospitalisation	804	21.2	<0.001	39	17.0	0.13
Included in a nutritional program	722	19.0	0.56	52	22.6	0.18
Deaths	56	1.5	0.41	7	3.0	0.06

* Comparison with children with watery diarrhea included in the surveillance study but not included in the bacteriology study

^‡^ Comparison between children with watery or bloody diarrhea

### Proportion of pathogenic bacteria, seasonal distribution and clinical characteristics

Overall, at least one pathogenic bacterium was found in 1060 (28.0%) children with watery diarrhea and 97 (42.2%) children with bloody diarrhea. EPEC, *Salmonella* spp. and *Campylobacter* spp. were similarly frequent in children with watery and bloody diarrhea and *Shigella* spp. were the most frequent among children with bloody diarrhea (Table 2). Mixed infections (i.e. association with rotavirus or any other bacteria) were found in 10.3% of children overall. Fifty-four children (1.3%) with sterile stool culture after 48h were excluded from all the subsequent analyses.

**Table 2:** Weighted proportion of enteric bacterial pathogens and rotavirus among children with watery and bloody diarrhea.

	**Watery diarrhea**			**Bloody diarrhea**		
	n	%	95% CI	n	%	95% CI
All						
Putative EPEC*	416	11.1	[10.1–12.2]	20	9.4	[6.1–13.2]
*Salmonella* spp.	340	9.2	[8.2–10.3]	20	8.7	[5.8–13.4]
*Shigella* spp.	78	2.0	[1.6–2.5]	50	22.1	[17.1–28.1]
*Campylobacter* spp. ^‡^	350	11.4	[10.2–12.6]	20	8.8	[5.7–13.3]
Rotavirus	1192	33.8	[32.2–35.5]	15	6.2	[3.7–10.1]
Mixed infections excluded						
Putative EPEC*	289	7.5	[6.6–8.4]	10	4.3	[2.3–7.9]
*Salmonella* spp.	184	4.8	[4.1–5.5]	15	6.7	[4.0–10.9]
*Shigella* spp.	54	1.3	[1.0–1.7]	42	18.2	[13.6–23.8]
*Campylobacter* spp. ^‡^	178	4.9	[4.2–5.7]	11	4.8	[2.7–8.5]
Rotavirus	917	26.2	[24.7–27.8]	10	4.2	[2.2–7.7]

* Including all isolates identified as putative EPEC in Maradi

^‡^ Analysis restricted to July 2010-March 2012

When the weighted proportions were extrapolated to the number of diarrhea cases, the seasonal distribution of pathogens showed a consistent peak of EPEC in March to June, when 54% of EPEC diarrhea occurred annually. There was a peak of *Salmonella* spp. in October to January during the first year with 50% of all *Salmonella* diarrhea (mean 27 cases per month). This same amplitude was not found the second year (mean 13 cases per month) (S1 Fig.).

**Fig 1:**
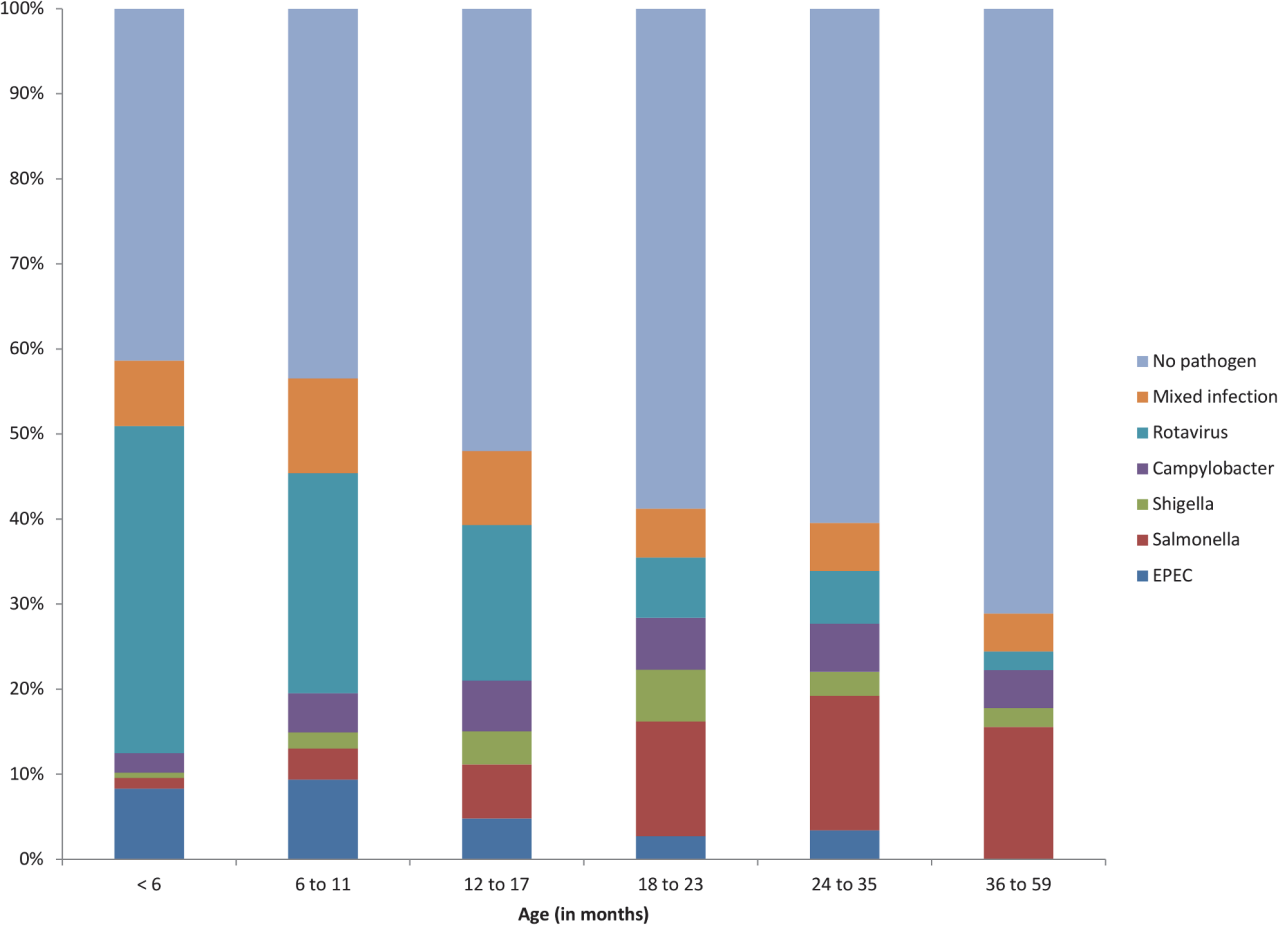
Distribution of organism identified in children with watery or bloody diarrhea by age group.

While the proportion of EPEC and rotavirus decreased with age, the proportion of *Salmonella* spp. increased (Fig. 1). All demographic and clinical characteristics showed significant differences among infection categories with the exception of sex (Table 3). The main associations after exclusion of mixed-infections were the following: *Campylobacter* infections with moderate dehydration (p = 0.007); EPEC infections with vomiting (p = 0.011) and severe dehydration (p<0.001); *Salmonella* infections with severe dehydration (p<0.001), fever (p = 0.016) and inclusion in a nutrition program (p<0.001); *Shigella* infections with deaths (p = 0.003); finally rotavirus infections with vomiting (p<0.001) and with non-inclusion in a nutrition program (p<0.001), as described elsewhere [32]. While all isolated *Campylobacter* were *C*. *jejuni*, diversity of the other pathogens is described below.

**Table 3:** Demographic and clinical characteristics of children with watery or bloody diarrhea by agent identified from stools, after exclusion of mixed infections.

	**No pathogen**	***Campylobacter* spp.**	**EPEC spp.**	***Salmonella* spp.**	***Shigella* spp.**	**Rotavirus**	** p**
	N = 1882	N = 189	N = 299	N = 199	N = 96	N = 928	
Sex, males, % (95% CI)	54.8 (52.5–57.0)	57.1 (50.0–64.2)	56.2 (50.6–61.8)	57.3 (50.4–64.2)	56.3 (46.3–66.2)	55.3 (52.1–58.5)	0.98
Age in months, mean (SD)	11.8 (7.0)	12.0 (6.0)	8.8 (4.1)	15.2 (7.7)	13.7 (6.7)	8.6 (4.2)	<0.001
N° of stools in the last 24h, mean (SD)	6.1 (1.6)	6.3 (1.5)	6.1 (1.7)	6.3 (1.6)	6.6 (1.5)	6.5 (1.6)	<0.001
Vomiting, % (95% CI)	55.0 (52.7–57.2)	54.8 (47.7–61.9)	69.9 (64.7–75.1)	52.7 (45.8–59.7)	48.9 (38.8–59.1)	82.1 (79.7–84.6)	<0.001
Severe dehydration, % (95% CI)	14.9 (13.3–16.6)	8.5 (4.5–12.5)	24.4 (19.5–29.3)	24.1 (18.2–30.1)	19.8 (11.8–27.8)	12.5 (10.4–14.6)	<0.001
Fever, % (95% CI)	22.8 (20.9–24.7)	23.3 (17.2–29.3)	23.4 (18.6–28.2)	29.1 (22.8–35.5)	29.1 (22.8–35.5)	20.2 (17.6–22.8)	0.07
Vesikari score, mean (SD)	11.8 (2.5)	11.7 (2.6)	12.6 (2.5)	12.1 (2.6)	11.4 (2.5)	12.7 (2.0)	<0.001
Death, % (95% CI)	1.5 (0.9–2.0)	1.1 (0.0–2.5)	2.3 (0.6–4.1)	2.0 (0.1–4.0)	5.2 (0.7–9.7)	0.9 (0.3–1.5)	0.031
In nutritional program	21.5 (19.6–23.3)	17.5 (12.0–22.9)	20.1 (15.5–24.6)	29.6 (23.3–36.0)	27.1 (18.1–36.0)	12.2 (10.1–14.3)	<0.001

### *Shigella* and *Salmonella* diversity

Of 143 isolates initially identified as *Shigella*, 138 were sent to the FNRC-ESS. The missing isolates were due to storage difficulties after strain identification. Ten (10) strains were not confirmed as *Shigella* spp., leaving 128 confirmed *Shigella* isolates. Of these, 6 were not serotypable (four were rough and two were non-agglutinable with the panel of typing antisera). Of the 122 serotyped isolates, the most frequent serogroups were *S*. *flexneri* (n = 69, 56.5%), followed by *S*. *dysenteriae* (n = 24, 19.7%), *S*. *boydii* (n = 21, 17.2%), and finally *S*. *sonnei* (n = 8, 6.6%). Two isolates belonged to *S*. *dysenteriae* type 1, the causative agent of epidemic dysentery. Details of serotypes identified are provided in Table 4.

**Table 4:** *Shigella* serotypes identified in the study.

***Shigella* serotypes**	**n**	**%**
*S*. *flexneri*	69	56.5
*S*. *flexneri* 1b	17	
*S*. *flexneri* 2a	17	
*S*. *flexneri* 6 Boyd 88	8	
*S*. *flexneri 2*b	7	
*S*. *flexneri* 3a	7	
*S*. *flexneri* 4	5	
*S*. *flexneri* Y	5	
*S*. *flexneri* 1c	2	
*S*. *flexneri 3*b	1	
*S*. *dysenteriae*	24	19.7
*S*. *dysenteriae* 2	16	
*S*. *dysenteriae* 3	4	
*S*. *dysenteriae* 1	2	
*S*. *dysenteriae* 12	2	
*S*. *boydii*	21	17.2
*S*. *boydii* 12	9	
*S*. *boydii* 1	3	
*S*. *boydii 2*	3	
*S*. *boydii 5*	2	
*S*. *boydii 3*	1	
*S*. *boydii 4*	1	
*S*. *boydii* 10	1	
*S*. *boydii* 18	1	
*S*. *sonnei*	8	6.6
*S*. *sonnei g*	8	
**Total** *	**122**	**100**

*Not including the four rough (auto-agglutinable) and the two non-agglutinable isolates

Of the 383 isolates initially identified as *Salmonella*, 370 were sent to the FNRC-ESS. The missing isolates were due to storage difficulties after strain identification. Nine (9) strains were not confirmed as *Salmonella* spp., one could not be subcultured, leading to 360 confirmed *Salmonella* isolates. In addition, 7 were not serotypable (rough). Two cultures contained more than one serotype; a mixture of serotypes Serenli and Suelldorf in one and a mixture of serotypes Mbao and Herston in the other. Among the 355 *Salmonella* serotypes, the most frequently encountered were Typhimurimum (n = 71, 20.0%), Enteritidis (n = 56, 15.8%), and Corvallis (n = 46, 13.0%) (Table 5). Another 64 serotypes were identified with less than 20 isolates for each, including *S*. *enterica* serotype Typhi (two isolates), the agent of typhoid fever and two potential new serotypes belonging to subspecies *enterica*. These potential new serotypes with antigenic formulae 44:z:z6 (one isolate) and 47:d:1,5 (two isolates) were sent to the World Health Organization Collaborative Center for Reference and Research on *Salmonella*, Institut Pasteur, Paris for confirmation and incorporation into the White-Kauffmann-Le Minor scheme. One with antigenic formula 47:d:1,5 has be confirmed and named serotype Maradi, while the other is still under study.

**Table 5:** *Salmonella* serotypes identified in the study (N = 355).

**Subspecies**	**Serotypes**	**n**	**Subspecies**	**Serotypes**	**n**
I.	Aberdeen	2	I.	Mbao	5
I.	Adelaide	5	I.	Menston	1
I.	Agona	1	I.	Mesbit	1
I.	Ank	2	I.	Molade	1
I.	Antsalova	1	I.	Montevideo	4
I.	Banana	11	I.	Muenchen	2
I.	Belem	1	I.	Muenster	4
I.	Brandenburg	1	I.	Ona	1
I.	Carmel	2	I.	Oranienburg	4
I.	Cerro	4	I.	Poona	6
I.	Chester	3	I.	Rissen	1
I.	Colindale	7	I.	Rubislaw	3
I.	Corvallis	46	I.	Saintpaul	1
I.	Denver	2	I.	Serenli	2
I.	Derby	1	I.	Stanleyville	1
I.	Ealing	1	I.	Suelldorf	1
I.	Eastbourne	1	I.	Telelkebir	1
I.	Elisabethville	1	I.	Tennessee	4
I.	Enteritidis	56	I.	Tilene	3
I.	Evry	1	I.	Typhi	2
I.	Freetown	2	I.	Typhimurium	71
I.	Gaminara	3	I.	Urbana	2
I.	Give	8	I.	Vejle	7
I.	Gombe	1	I.	Virchow	1
I.	Hadar	1	I.	Waycross	1
I.	Hato	2	I.	Wimborne	4
I.	Havana	6	I.	Zinder	3
I.	Herston	1	I.	Provisional new serotype I 44:z:z6	1
I.	Hull	1	I.	New serotype Maradi (47:d:1,5)	2
I.	Johannesburg	15	I.	1,3,19:d:- (monophasic)	3
I.	Kalina	2	I.	4,5,12:-:1,2 (monophasic)	1
I.	Kentucky	2	I.	4,5,12:i:- (monophasic)	4
I.	Maastricht	12	IIIa.	41:z4,z23:-:-	1
I.	Magherafelt	1			

I., subspecies *enterica*; IIIa, subspecies *arizonae*; the 7 isolates (6 belonging to subsp. *enterica* and one to subsp. *salamae*) with a rough phenotype were not included.

### Characterization of EPEC isolates

Of the 436 isolates initially screened as putative EPEC isolates by O serogrouping, a random sample of 74 isolates selected from rotavirus-negative children and all 37 isolates from rotavirus-positive children isolated in 2010 were characterized at the FNRC-ESS. Of these 111 isolates, 104 (93.7%) were confirmed as *E*. *coli* based on biochemical and phenotypic reactions, and 90/104 (86.5%) were confirmed to be EPEC due to the presence of the *eae* gene encoding intimin and the absence of other EHEC-associated virulence genes, *stx1*, *stx2*, and EHEC-*hlyA* (S2 Table). Of the 90 EPEC, 88 (97.8%) were typical EPEC due to the presence of the *bfpA* gene carried by the EAF plasmid. Of the 14 other *E*. *coli*, 6 were actually EAEC due to the presence of the *aggR* gene and 8 did not contain any of the targeted virulence genes and were probably not pathogenic *E*. *coli*. The most frequent EPEC serogroups were O55 (32/90, 35.5%), O126 and O119 (13/90, 14.4% each) (S2 Table). All but two EAEC also belonged to O55. Four of the 8 isolates without virulence genes belonged to serogroups common to EPEC such as O26, O114 and O126. There was no significant difference in the proportion of intimin-positive isolates among *E*. *coli* isolated in rotavirus-positive or negative children (p = 0.37).

### Antimicrobial resistance

Around 2% (6/328) of *Campylobacter jejuni* were resistant to amoxicillin-clavulanic acid and erythromycin, with 5 isolates resistant to both. More than half of all Enterobacteriaceae, and up to 90% of EPEC isolates were resistant to amoxicillin and co-trimoxazole, while they were mostly sensitive to all other antibiotics tested (Table 6). Among *Shigella* isolates, *S*. *flexneri* (71.8%) were more resistant to amoxicillin than *S*. *sonnei* (12.5%), but similarly resistant to co-trimoxazole (86.0% in *S*. *flexneri* and 100% in *S*. *sonnei*). The two *S*. *dysenteriae* type 1 isolates were resistant to amoxicillin and susceptible to other tested drugs. Forty-six *Salmonella* and one EPEC isolate were resistant to ESCs and exhibited an ESBL phenotype. All the 46 ESBL-producing *Salmonella* belonged to serotype Corvallis. The majority of these isolates were also resistant to gentamicin (n = 31) and intermediate or resistant to ciprofloxacin (n = 23, MICs range 1–3 mg/L). Although there was a peak of ESBL-producing *S*. *enterica* serotype Corvallis in November 2010 (n = 13), the others were isolated throughout the study period. The district of origin of children with ESBL-producing *Salmonella* infection was more likely to be Maradi (41.3%, n = 19) compared to children with any *Salmonella* infection (12.0%, p<0.001). However, ESBL-producing *S*. *enterica* serotype Corvallis were also isolated from Guidan-Roumdji (n = 13), Madarounfa (n = 9) and other districts. Regarding resistance to ciprofloxacin, in addition to Corvallis isolates, only two other isolates were resistant (MICs 16 mg/L). Both isolates belonged to serotype Kentucky and were ST198 by MLST.

**Table 6:** Antibiotic resistance of EPEC, *Salmonella* spp. and *Shigella* spp. isolated in the study.

	**EPEC (N = 434)**				***Salmonella* (N = 360)**				***Shigella* (N = 128)**			
	**Intermediate**		**Resistant**		**Intermediate**		**Resistant**		**Intermediate**		**Resistant**	
	n	%	n	%	n	%	n	%	n	%	n	%
Amoxicillin	1	0.2	386	88.9	0	0.0	186	51.7	1	0.8	79	61.7
Amoxicillin-clavulanic acid	114	26.3	9	2.1	20	5.6	45	12.5	15	11.7	2	1.6
Cefotaxime	0	0.0	1	0.2	0	0.0	46	12.8	0	0.0	0	0.0
Ceftazidime	0	0.0	1	0.2	0	0.0	46	12.8	0	0.0	0	0.0
Cefepime	0	0.0	1	0.2	0	0.0	46	12.8	0	0.0	0	0.0
Imipeneme	0	0.0	0	0.0	0	0.0	0	0.0	0	0.0	0	0.0
Nalidixic acid	0	0.0	7	1.6	9	2.5	21	5.8	0	0.0	0	0.0
Ciprofloxacin	1	0.2	1	0.2	6	1.7	23	6.4	0	0.0	0	0.0
Gentamicin	0	0.0	2	0.5	0	0.0	33	9.2	0	0.0	1	0.8
Amikacin	0	0.0	1	0.2	1	0.3	3	0.8	0	0.0	0	0.0
Co-trimoxazole	0	0.0	419	96.5	0	0.0	180	50.0	0	0.0	110	85.9

## Discussion

Almost one-third of children were infected with or carried a bacterial pathogen and around one in ten presented co-infections with either another bacterial pathogens or rotavirus, consistent with what has been found in other African countries [9,12]. Although it is widely recognized that isolation of a bacterial pathogen from stool does not directly imply that the diarrhea is attributable to this particular pathogen, the characterization of circulating agents in a population still provides important information for the prevention and control of these infections and can complement case-control studies on diarrhea etiology. In case-control studies, rotavirus and *Shigella* spp. are consistently reported as highly associated with diarrhea patients (cases), EPEC shows significant but intermediate association, while *Salmonella* spp. and *Campylobacter* are often found in similar proportions in patients with or without diarrhea [7,50,51]. Here, it is thus difficult to conclude on the fractions of diarrhea cases attributable to each pathogen, except for *Shigella* spp., which is probably responsible for around 2% of non-bloody diarrhea and over 20% of bloody diarrhea in this area.

In our study, *Shigella* spp. were associated with the highest mortality overall and particularly affected children included in a nutritional program, with a case fatality ratio of 15%. Most *Shigella* spp. isolates were resistant to amoxicillin, although *S*. *sonnei* was mostly sensitive, similar to what was described in Senegal and Central African Republic [23,52]. Resistance to ESCs and fluoroquinolones was not found here, which is also consistent with the low prevalence in other reports from Africa and in contrast with the emergence of resistance in Asia [9,52–55]. The isolated *Shigella* serotypes showed the typical pattern described in developing countries with a clear predominance of *S*. *flexneri*, in particular over *S*. *sonnei* [56]. Several Asian countries have recently experienced a transition from predominance of *S*. *flexneri* to *S*. *sonnei*, probably due to improvement of overall nutritional status, sanitation and socioeconomic status [57–59]. The few studies available from sub-Saharan Africa suggest that it is not yet the case in this area [9,52,53,60], which emphasizes the need for vaccines including both species.

The isolation of *S*. *dysenteriae* type 1 from two children living in two different areas and 2 months apart indicates that this pathogen was still circulating in this area in 2011 but represented only 1.6% of all *Shigella* spp. and was not associated with outbreaks. The low level circulation of *S*. *dysenteriae* type 1 was also observed in a survey conducted in an urban slum in Nairobi, Kenya between 2007 and 2010 where *S*. *dysenteriae* type 1 represented 2.3% (6/262) of *Shigella* spp. isolated [53]. Although no outbreak due to *S*. *dysenteriae* type 1 has been reported in Africa since 2003–2004 [61], these studies document the low level circulation of Shiga’s bacillus in two different regions of Africa where circumstances facilitating its transmission such as overcrowding, poor or lack of sanitation or health facilities might trigger local outbreaks.

Although screening for putative EPEC was based on O antigens (serogrouping) rather than on the presence of virulence genes, the *eae* gene, marker of EPEC was found in the vast majority of isolates, confirming the good correlation between serogroups and virulence. However, the identification method itself may be a limiting factor because it did not allow for the identification of other *E*. *coli* enteric pathovars, and in particular ETEC, which was identified as the cause of one third of the diarrhea attributable fraction (and first bacterial agent) in the GEMS study [7]. EAEC were also found as the most frequent bacterial pathogens in children with diarrhea in Africa, particularly in infants [9,12,62]. In our study, EPEC was associated with younger age, as well as vomiting and severe dehydration. It was not associated with a higher risk of dying, even in children less than 1 year (p = 0.11), in contrast to the 2.6 hazard ratio found in GEMS [7]. EPEC isolates presented the highest resistance to amoxicillin and co-trimoxazole, but were mostly sensitive to other antibiotics tested. The detection of one ESBL-producing EPEC warrants attention in the context of increasing resistance in Africa [29,63].

We found that almost all EPEC (97.8%) were typical in agreement with other published data [64]. Typical EPEC are associated with a human reservoir in low-income countries whereas atypical EPEC are closer to Shiga toxin-producing *E*. *coli* and can be found either in humans or animals of high-income countries. Interestingly, we identified typical EPEC belonging to a new serogroup or to a serogroup (O26) previously associated only with atypical EPEC [64]. This could be due to a large diversity of EPEC populations circulating in low-income countries and not well-known due to the lack of local studies requiring molecular biology for EPEC confirmation.

*Salmonella* spp. were more frequently isolated in children included in a nutrition program, the proxy used to define the population of malnourished children in our study. This suggests that malnutrition could be a risk factor for *Salmonella* infection in general, and not only for invasive non-typhoidal *Salmonella* infection [17]. The *Salmonella* peak around November 2010 corresponds to end of the annual malnutrition peak in Niger, and the number of children included in a nutritional program during this period in our study was decreasing (data not shown), suggesting that the 2 phenomena are temporally disconnected. This period also generally corresponds to the malaria peak in Niger, but we could not study possible associations between malaria and *Salmonella* isolation in diarrhea patients here since diagnosis of malaria was not recorded. In addition, although there was a slight increase in the number of *Salmonella* detected around November 2011, there was no clear peak and the seasonality of infection remains to be confirmed.

Despite a wide variety of *Salmonella* serotypes identified, around half of the isolates belonged to serotypes Typhimurium, Enteritidis and Corvallis. While the 2 first are known to be involved in a majority of diarrhea, as well as invasive NTS infections in Africa [17,65–67], serotype Corvallis has not been described as a major serotype so far. The fact that almost all serotype Corvallis isolates showed very similar antimicrobial resistance profiles, including ESBL production, suggests that there was a clonal strain spreading in the area. This spread was disseminated, in both space and time since it was found in all districts and during the whole study period, with a peak in November 2010, along with other *Salmonella* spp. The comprehensive characterization of these isolates as well as others from older and more recent surveys is ongoing in order to elucidate the reservoir of this bacterial population. Two isolates of serotype Kentucky highly resistant to ciprofloxacin were isolated. They belonged to the multi-drug resistant *S*. *enterica* serotype Kentucky ST198-X1 strain which originated in Egypt during the 1990s, before disseminating across Africa, the Middle East, the Indian subcontinent and South East Asia beginning in the mid-2000s [37,68].

Our study had several limitations. As mentioned above, the study was not designed to assess attributable fractions and it is difficult to conclude on the precise etiology of diarrhea in this context. Since the required case-control studies for such a purpose are time- and resource-consuming, knowledge of pathogen and site-specific odds ratio from multicentric studies such as GEMS would contribute to the interpretation of descriptive studies. In addition, the spectrum of bacterial pathogens sought did not include some important bacteria such as ETEC, EAEC, due to the lack of appropriate techniques (ie. PCR) to detect them on site. EHEC O157:H7, which can be detected by culture using appropriate media, was also not investigated here since it was not isolated in a previous study in the same area [18]. Media for the detection of *V*. *cholerae* were available in case of suspicion of cholera, but were not used systematically.

In conclusion, this study provides updated information on enteric bacteria diversity and antimicrobial resistance in the Sahel region, where such data are scarce. Although rotavirus represents the first cause of diarrhea with dehydration, potentially pathogenic bacteria were found in a similar proportion of children (~30%), sometimes in co-infection. Whether they are or not the causative agent of diarrhea, bacterial infections and their antimicrobial resistance profiles should be considered in areas with a large proportion of malnutrition, which pre-disposes to severe and invasive infections. The emergence of ESBL-producing *Salmonella* spp. resistant to all locally available antimicrobials should particularly be monitored. The wide serotype diversity, in particular for *Shigella* spp., should also be considered for vaccine development.

## Supporting Information

S1 Dataset Dataset and codebook. (XLS)

S1 Fig Weighted proportion of Enteropathogenic *Escherichia coli* (EPEC), *Salmonella* spp., *Campylobacter* spp. and *Shigella* spp. by month. (TIF)

S1 Table Oligonucleotide primers used for targeting virulence genes. (DOCX)

S2 Table Distribution of the different pathotypes and serogroups among the selection of 104 putative Enteropathogenic *Escherichia coli* (EPEC) studied at the reference laboratory (FNRC-ESS). (DOCX)
