# Supplementary figures and images for: Enteric Bacterial Pathogens in Children with Diarrhea in Niger: Diversity and Antimicrobial Resistance
Source: PLoS One. 2015 Mar 23;10(3):e0120275. doi: 10.1371/journal.pone.0120275 (PMC4370739; doi:10.1371/journal.pone.0120275)

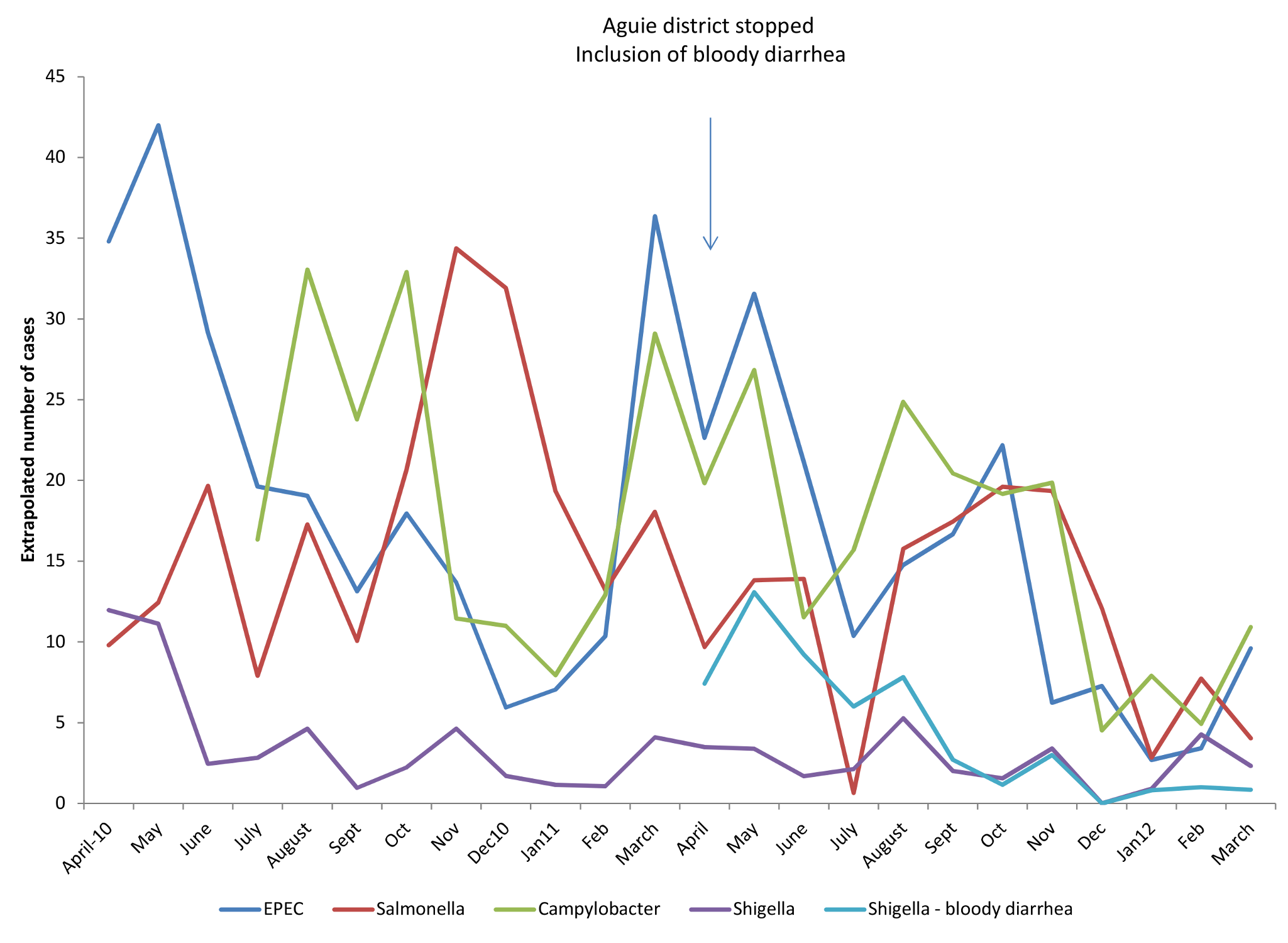

Supplement: S1 Fig — (TIF) [file pone.0120275.s002.tif]
