# Supplementary material for: Enteric Bacterial Pathogens in Children with Diarrhea in Niger: Diversity and Antimicrobial Resistance
Source: PLoS One. 2015 Mar 23;10(3):e0120275. doi: 10.1371/journal.pone.0120275 (PMC4370739; doi:10.1371/journal.pone.0120275)
Supplement: S1 Table — (DOCX) [file pone.0120275.s003.docx]

**S1 Table. Oligonucleotide primers used for targeting virulence genes.**

| **PCR target** | **Primer name** | **Oligonucleotide sequence (5’→3’)** | **Annealing temperature**  **(°C)** | **PCR product size (bp)** | **Reference** |
| --- | --- | --- | --- | --- | --- |
| *uidA* | UAL-754 | AAAACGGCAAGAAAAAGCAG | 43 | 147 | [38] |
|  | UAR-900 | ACGCGTGGTTACAGTCTTGCG |  |  |  |
| *eaeA* | fM1 | CATTATGGAACGGCAGAGGT | 60 | 790 | [39] |
|  | rYu4 | ATCTTCTGCGTACTGCGTTCA |  |  |  |
| *bfpA* | EP1 | AATGGTGCTTGCGCTTGCTGC | 56 | 326 | [40] |
|  | EP2 | GCCGCTTTATCCAACCTGGTA |  |  |  |
| *vt* | Lin 5’ | GAACGAAATAATTTATATGT | 43 | 900 | [41] |
|  | Lin 3’ | TTTGATTGTTACAGTCAT |  |  |  |
| *stx1* | Lin 5’ | GAACGAAATAATTTATATGT | 43 | 726 | [41,42] |
|  | VT1b | AGCGATGCAGCTATTAATAA |  |  |  |
| *stx2* | Lin 5’ | GAACGAAATAATTTATATGT | 43 | 504 | [41,43] |
|  | stx2-R | TCGCCAGTTATCTGACATTCTG |  |  |  |
| EHEC-*hlyA* | hlyAF | GCATCATCAAGCGTACGTTCC | 60 | 534 | [43] |
|  | hlyAR | AATGAGCCAAGCTGGTTAAGCT |  |  |  |
| *aggR* | AggR-F | GTATGAAATTAAAACAAACATCG | 50 | 800 | [44] |
|  | AggR-R | GTTTATTGGCTTTTAAAATAGTC |  |  |  |
| *st* | ST1F | ATTTTTCTTTCTGTATTGTCT | 53 | 183 | [45,46] |
|  | STa2 | ACAGGCAGGATTACAACAAAG |  |  |  |
| *LT* | LT1 | ATTTACGGCGTTACTATCCTC | 43 | 280 | [46] |
|  | LT2 | TTTTGGTCTCGGTCAGATATG |  |  |  |
| *ial* | PR1 | CTGGATGGTATGGTGAGG | 55 | 320 | [47] |
|  | PR2 | GGAGGCCAACAATTATTTCC |  |  |  |
| *ipaH* | ipaH14a | GTTCCTTGACCGCCTTTCCGATACCGTC | 55 | 620 | [38] |
|  | ipaH14b | GCCGGTCAGCCACCCTCTGAGAGTAC |  |  |  |
