# Supplementary material for: Enteric Bacterial Pathogens in Children with Diarrhea in Niger: Diversity and Antimicrobial Resistance
Source: PLoS One. 2015 Mar 23;10(3):e0120275. doi: 10.1371/journal.pone.0120275 (PMC4370739; doi:10.1371/journal.pone.0120275)
Supplement: S2 Table — (DOCX) [file pone.0120275.s004.docx]

**S2 Table. Distribution of the different pathotypes and serogroups among the selection of 104 putative Enteropathogenic *Escherichia coli* (EPEC) studied at the reference laboratory (FNRC-ESS)**

| **Serogroups** | ***E. coli* studied** | **Enteropathogenic *E. coli* (EPEC)** | | **Enteroaggregative *E. coli* (EAEC)** | | **Other^‡^** | |
| --- | --- | --- | --- | --- | --- | --- | --- |
|  | **N** | **n** | **%** | **n** | **%** | **n** | **%** |
| O55 | 36 | 32 | 88.9 | 4 | 11.1 | 0 | 0.0 |
| O126 | 14 | 13 | 92.9 | 0 | 0.0 | 1 | 7.1 |
| O119 | 13 | 13 | 100.0 | 0 | 0.0 | 0 | 0.0 |
| O142 | 8 | 8 | 100.0 | 0 | 0.0 | 0 | 0.0 |
| O127 | 7 | 7 | 100.0 | 0 | 0.0 | 0 | 0.0 |
| O125 | 7 | 7 | 100.0 | 0 | 0.0 | 0 | 0.0 |
| O26 | 5 | 4 | 80.0 | 0 | 0.0 | 1 | 20.0 |
| O86 | 2 | 2 | 100.0 | 0 | 0.0 | 0 | 0.0 |
| O114 | 2 | 0 | 0.0 | 0 | 0.0 | 2 | 100.0 |
| O128 | 1 | 1 | 100.0 | 0 | 0.0 | 0 | 0.0 |
| O161* | 2 | 0 | 0.0 | 2 | 100.0 | 0 | 0.0 |
| Non typable^#^ | 7 | 3 | 42.9 | 0 | 0.0 | 4 | 57.1 |
| **Total** | **104** | **90** | **86.5** | **6** | **5.8** | **8** | **7.7** |

*determined by *rfb*-RFLP

^#^*rfb*-RFLP typing only performed on the 3 EPEC isolates. They showed a similar new *rfb*-RFLP type not present in the FNRC-ESS database

^‡^*E. coli* that does not contain any of the virulence genes assayed
